# Blockade of ROCK inhibits migration of human primary keratinocytes and malignant epithelial skin cells by regulating actomyosin contractility

**DOI:** 10.1038/s41598-019-56447-2

**Published:** 2019-12-27

**Authors:** Srisathya Srinivasan, Sreya Das, Vishakha Surve, Ankita Srivastava, Sushant Kumar, Nikita Jain, Abhijeet Sawant, Chitra Nayak, Rahul Purwar

**Affiliations:** 1 Department of Biosciences & Bioengineering, IIT Bombay, Mumbai, Maharashtra India; 2 Department of Plastic Surgery, Topiwala National Medical College & BYL Nair Charitable Hospital, Mumbai, Maharashtra India; 3 Department of Skin and Venereal Diseases, Topiwala National Medical College & BYL Nair Charitable Hospital, Mumbai, Maharashtra India

**Keywords:** Cell migration, Myosin

## Abstract

Actomyosin contractility, crucial for several physiological processes including migration, is controlled by the phosphorylation of myosin light chain (MLC). Rho-associated protein kinase (ROCK) and Myosin light chain kinase (MLCK) are predominant kinases that phosphorylate MLC. However, the distinct roles of these kinases in regulating actomyosin contractility and their subsequent impact on the migration of healthy and malignant skin cells is poorly understood. We observed that blockade of ROCK in healthy primary keratinocytes (HPKs) and epidermal carcinoma cell line (A-431 cells) resulted in loss of migration, contractility, focal adhesions, stress fibres, and changes in morphology due to reduction in phosphorylated MLC levels. In contrast, blockade of MLCK reduced migration, contractile dynamics, focal adhesions and phosphorylated MLC levels of HPKs alone and had no effect on A-431 cells due to the negligible MLCK expression. Using genetically modified A-431 cells expressing phosphomimetic mutant of p-MLC, we show that ROCK dependent phosphorylated MLC controls the migration, focal adhesion, stress fibre organization and the morphology of the cells. In conclusion, our data indicate that ROCK is the major kinase of MLC phosphorylation in both HPKs and A-431 cells, and regulates the contractility and migration of healthy as well as malignant skin epithelial cells.

## Introduction

Keratinocyte is the predominant cell type present in the epidermis. Being the outer most layer of the skin, the epidermis is highly susceptible to injury and various environmental insults. Keratinocyte migration is an important physiological process not only during homeostasis (skin regeneration and wound healing) but is also critical in various pathologies (epidermal inflammation and malignancies)^1^. One of the most common causes of neoplastic transformation in keratinocytes is ultraviolet B (UVB) exposure^2^. This results in mutations in critical genes like p53, causing resistance to apoptosis in keratinocytes. This leads to uncontrolled proliferation and development of keratinocyte cancers like squamous cell carcinoma^3^. Unlike healthy keratinocytes, transformed keratinocytes are highly capable of invading the basement membrane, leading to metastasis^4^. The invasion of these transformed keratinocytes is dependent on several biochemical and biophysical cues in the tumour cell.

Contractility is one of the most important biophysical properties of the cell, which is critical for migration and invasion of healthy and malignant cells. Isoforms of myosin, namely non-muscle myosin IIA (NMMIIA), Non-muscle myosin IIB (NMMIIB) and Non-muscle myosin IIC (NMMIIC), are crucial for maintaining the contractility and migration of cells^5^. Though NMMIIB and NMMIIC have been implicated in cell migration and contractility, NMMIIA is known to be responsible for the generation of almost 60% of the traction forces in the cell^6^. NMMIIA is an actin dependent motor and acts as a master regulator of cellular contractility^7^. The regulation of NMMIIA function is dependent on phosphorylation of the myosin light chain (MLC), mainly regulated by Myosin light chain kinase (MLCK) and Rho-dependent protein kinase (ROCK)^8^. MLCK exclusively phosphorylates MLC, whereas ROCK phosphorylates MLC as well as regulates the activity of myosin phosphatase, an enzyme that dephosphorylates MLC^9^. Several studies have shown that the increased plasticity of cancer cells allows them to regulate their contractility and migration by altering p-MLC levels^10^, levels of MLCK^11^, or ROCK^12^.

Though ROCK and MLCK are the major kinases that phosphorylate MLC in migrating cells, their function is seen to be highly cell type specific^8^. Several studies have shown contrasting effects of MLCK or ROCK blockade in cells of various origins. While we and others have shown that blockade of ROCK by Y-27632 severely impairs the contractility and migration of malignant epithelial cells^13,14^, other studies have shown that Y-27632 promotes the proliferation and migration of corneal endothelial cells^15^ and limbal epithelial cells^16^. ROCK blockade by Y-27632 reduced the metastasis of breast cancer cells to bone in a mouse model^17^, and decreased the invasion of CaOV3 and SCOV3 ovarian cancer cell lines^18^, A-549 lung adenocarcinoma cells^19^ and small cell lung cancer cells^20^. In contrast, ROCK inhibition led to the activation of non-metastatic MCF-7 cells, resulting in increased invasion in a 3D substrate^21^ and increased invasion of SW480 colon cancer cells by altering focal adhesion dynamics^22^. Similarly, MLCK inhibition led to a decrease in invasion of pancreatic cancer cells^23^, and contrastingly, MLCK dependent cytoskeletal reorganization is also known to promote angiogenesis, a key step in cancer progression^24^. It seems evident that ROCK and MLCK are of paramount importance for cell migration, and their effect depends on the origin and type of cell studied. Identifying the pathways that govern actomyosin machinery in different cell types is critical for designing alternative therapies which can target the key cell specific mediators of these processes.

Although many studies have evaluated the ROCK as a therapeutic target for invasion and metastasis using ROCK specific inhibitors (small molecules), the distinct roles of ROCK and MLCK in regulating NMMIIA activity and their subsequent effects on migration of healthy and malignant human keratinocytes remains unknown. In this study, we demonstrate that the actomyosin machinery and the 3D migration of human primary keratinocytes (HPKs) and malignant epidermoid carcinoma cells (A-431) are differentially regulated by ROCK and MLCK. Using specific small molecule inhibitors as well as genetic manipulation techniques, we show that the ROCK pathway is crucial for regulating the contractility, focal adhesion dynamics, actin stress fibre organization and ECM remodelling ability of both HPKs and A-431 cells.

## Results

### ROCK is crucial for the migration of HPKs and A-431

**Figure 1:**
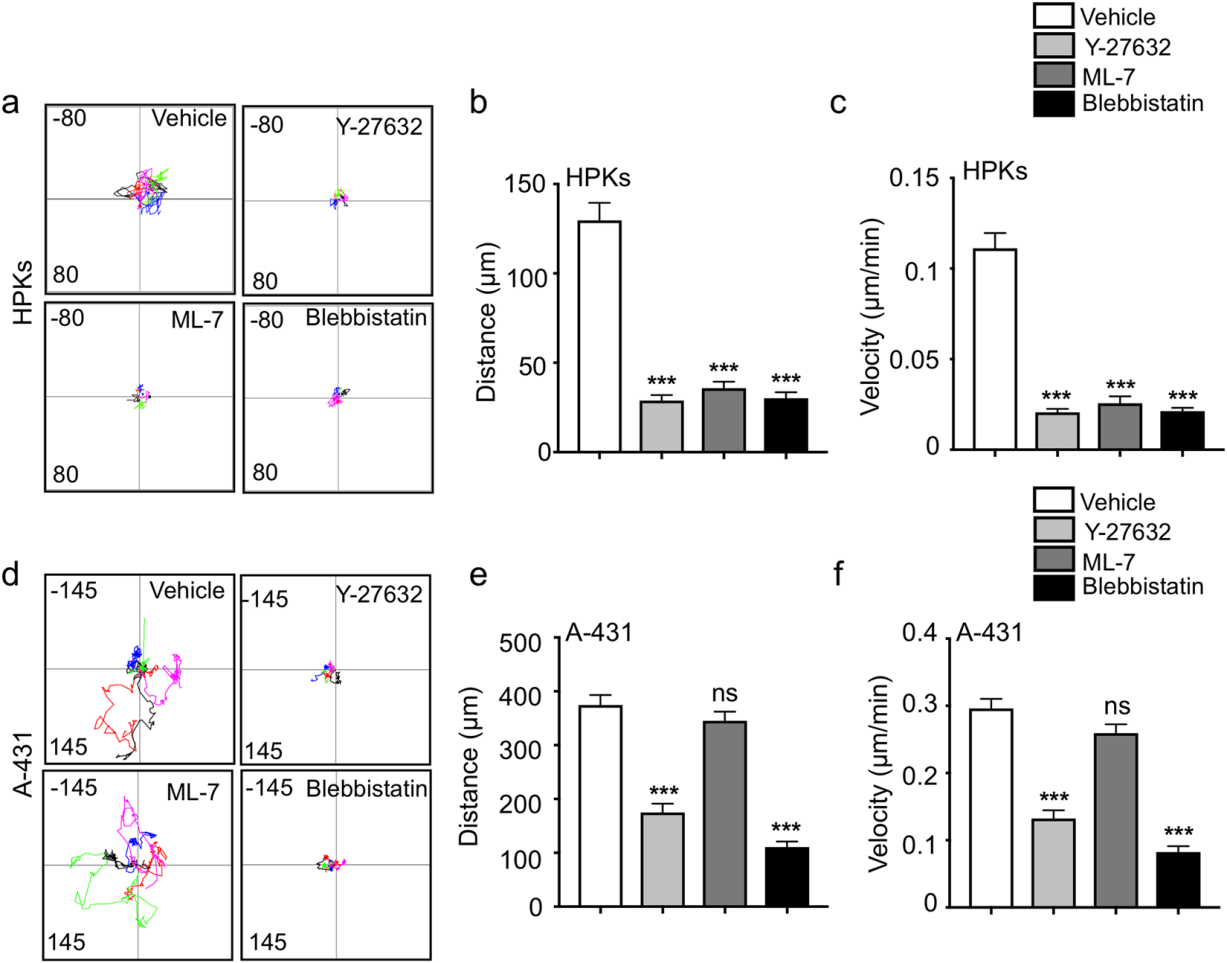
Roles of ROCK and MLCK on the migration potential of HPKs and A-431 cells. (**a**) Representative plots of migration of the HPKs in a 3D gel treated with the inhibitors. (**b**,**c**) Quantification of the total distance traversed (**b**) and the velocity (**c**) of HPKs in a 3D collagen gel. n = 3. (**d**) Representative track plots of invasion of the A-431 cells in a 3D gel treated with the inhibitors. (**e**,**f**) Quantification of the total distance traversed (**e**) and the velocity (**f**) of A-431 in a 3D collagen gel. n = 3. Data is represented Mean + SEM for bar graphs. Standard student’s t-test (two tailed) was performed ***p < 0.001, ns: not significant.

As a first step towards understanding the role of ROCK and MLCK in the migration of healthy and malignant keratinocytes, we examined the effect of inhibition of ROCK and MLCK on the migration of HPKs and A-431 by using a physiologically relevant 3-dimensional (3D) collagen gel. Y-27632 was used as a ROCK inhibitor, ML-7 as the MLCK inhibitor and Blebbistatin was used for global myosin inhibition. The inhibitors were used at a concentration where they were specific towards their targets and did not result in cell death or cell detachment^13,25,26^. The migration of HPKs and A-431 in the collagen gel was examined over a period of 24 hours by live cell imaging in the presence of inhibitors. There was a significant reduction in the migration upon ROCK inhibition in both HPKs (Fig. 1a–c) and A-431 (Fig. 1d–f), resulting in a decrease in both the distance traversed and the velocity of the cells. Inhibition of MLCK reduced the migration of HPKs but not A-431 (Fig. 1a–f). This suggests that ROCK is crucial for the 3D migration of both HPKs and A-431 cells. Though MLCK regulates the 3D migration of HPKs, it has no impact on the 3D migration of A-431 cells.

### Blockade of ROCK reduces the cellular contractility of both A-431 and HPKs

**Figure 2:**
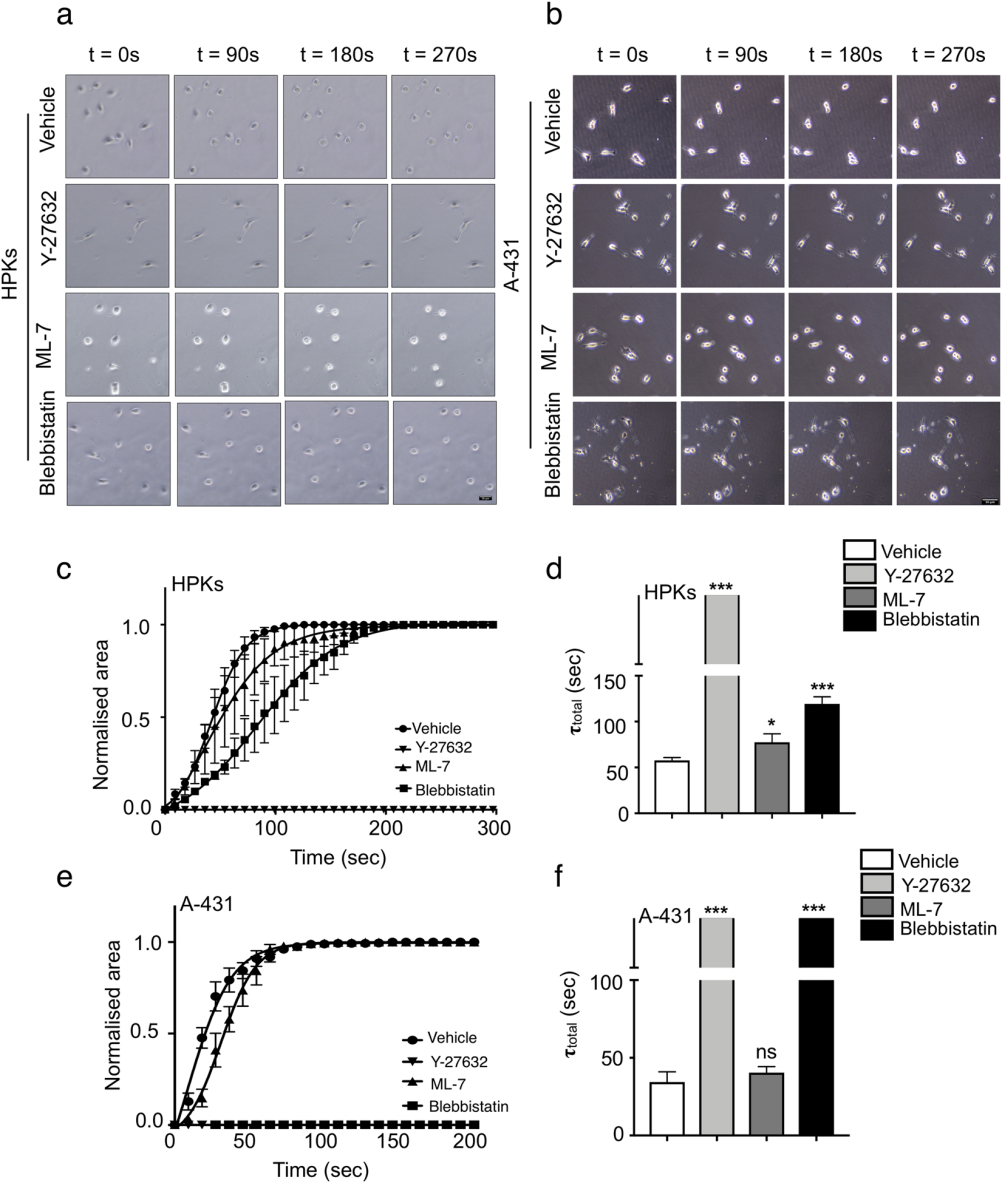
Effect of ROCK and MLCK on the contractility of HPKs and A-431 cells. (**a**,**b**) Representative time lapse images of the deadhesion dynamics of HPKs (**a**) and A-431 (**b**). Magnification 10X. Scale bar 50 μm. (**c**) Quantification of the deadhesion dynamics of HPKs treated with inhibitors. Normalized change in area was fitted using Boltzmann sigmoidal equation to give the time constants **τ**_1_ and **τ**_2_. (**d**) Quantification of **τ**_total_ of HPKs calculated as the sum of **τ**_1_ and **τ**_2._ n = 6. (**e**) Quantification of the deadhesion dynamics of A-431 cells treated with inhibitors. (**f**) Quantification of **τ**_total_ of A-431 calculated as the sum of **τ**_1_ and **τ**_2._ n = 3. Line graph is represented as Mean ± SEM, bar graphs are represented as Mean + SEM. Standard student’s t-test (two tailed) was performed *p < 0.05, ***p < 0.001, ns: not significant.

Cellular contractility is a crucial property and several fundamental cellular processes, including migration, depend on the ability of the cell to exert contractile force. Since we observed that ROCK and MLCK have distinct roles in regulating the migration of HPKs and A-431, we examined the roles of ROCK and MLCK on the retraction dynamics of HPKs and A-431 cells using the trypsin deadhesion assay^27^. The cells were treated with inhibitors and the retraction of the cells was analysed by time lapse microscopy (Fig. 2a,b). Highly contractile cells are expected to retract much faster as compared to less contractile cells. We observed that inhibition of ROCK by Y-27632 resulted in complete loss of cell retraction in both HPKs (Fig. 2c,d) and A-431 (Fig. 2e,f), as evident by the delayed time taken by the cells to retract in the presence of trypsin, indicating disruption of the contractility of both the cell types entirely. Blockade of MLCK resulted in a loss of contractility in HPKs, though no effect was observed in the contractility of A-431 cells. Blebbistatin treatment significantly inhibited the contractility in HPKs and A-431 cells, similar to Y-27632 treatment (Fig. 2c–f). These data suggest that ROCK activity is crucial for maintaining the contractility of both HPKs and A-431 cells. MLCK plays a role in regulating contractility in HPKs, but not in A-431 cells.

### ROCK controls focal adhesion maturation of HPKs and A-431 cells, whereas MLCK regulates focal adhesion localization and maturation of HPKs alone

**Figure 3:**
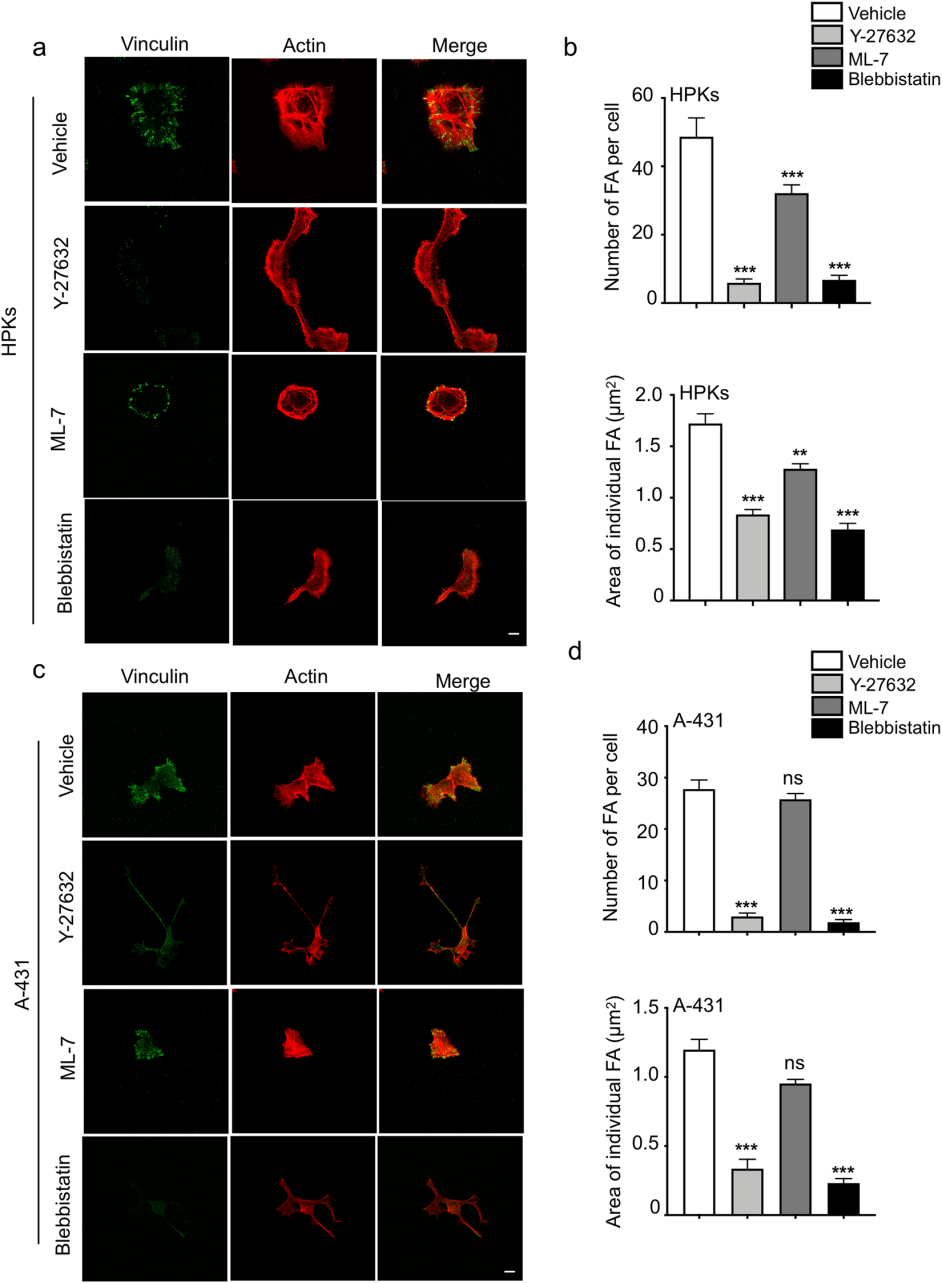
Regulation of focal adhesion organization by ROCK and MLCK in HPKs and A-431. (**a**) Representative images of vinculin (green) and actin (red) of HPKs. Magnification 63X. Scale bar 10 μm. (**b**) Quantification of the number and average area of the focal adhesions in HPKs treated with inhibitors. n = 3. (**c**) Representative images of vinculin (green) and actin (red) of A-431. Magnification 63X. Scale bar 10 μm. (**d**) Quantification of the number and average area of the focal adhesions in A-431 treated with inhibitors. n = 3. Data is represented Mean + SEM for bar graph. Standard student’s t-test (two tailed) was performed **p < 0.01, ***p 0.001, ns: not significant.

Focal adhesion formation is a myosin driven process and is crucial for migration and cell adhesion. Focal adhesions help convert the contractile forces generated in the cell into traction forces, which helps to pull the cells in the direction of motion^28^. Since we observed that ROCK and MLCK have distinct functions in regulating the contractility of HPKs and A-431, we examined the roles of these kinases on the focal adhesion organization of HPKs and A-431. ROCK blockade by Y-27632 resulted in a significant reduction in both the number and area of focal adhesions in both HPKs (Fig. 3a,b) and A-431 cells (Fig. 3c,d), indicating a complete loss in mature focal adhesions. Blockade of MLCK by ML-7 reduced the number and size of focal adhesions as well as localized the focal adhesions to the periphery of the HPKs (Fig. 3a,b), with no impact on focal adhesion organization in A-431 cells (Fig. 3c,d). In both the cell types, treatment with Blebbistatin resulted in a loss in both number and size of the focal adhesions, similar to Y-27632 treatment (Fig. 3a–d). These data indicate that ROCK is the major kinase that regulates the formation of mature focal adhesions in HPKs and A-431 cells.

### ROCK regulates actin stress fibres formation in both HPKs and A-431, and MLCK regulates stress fibre formation in only HPKs

**Figure 4:**
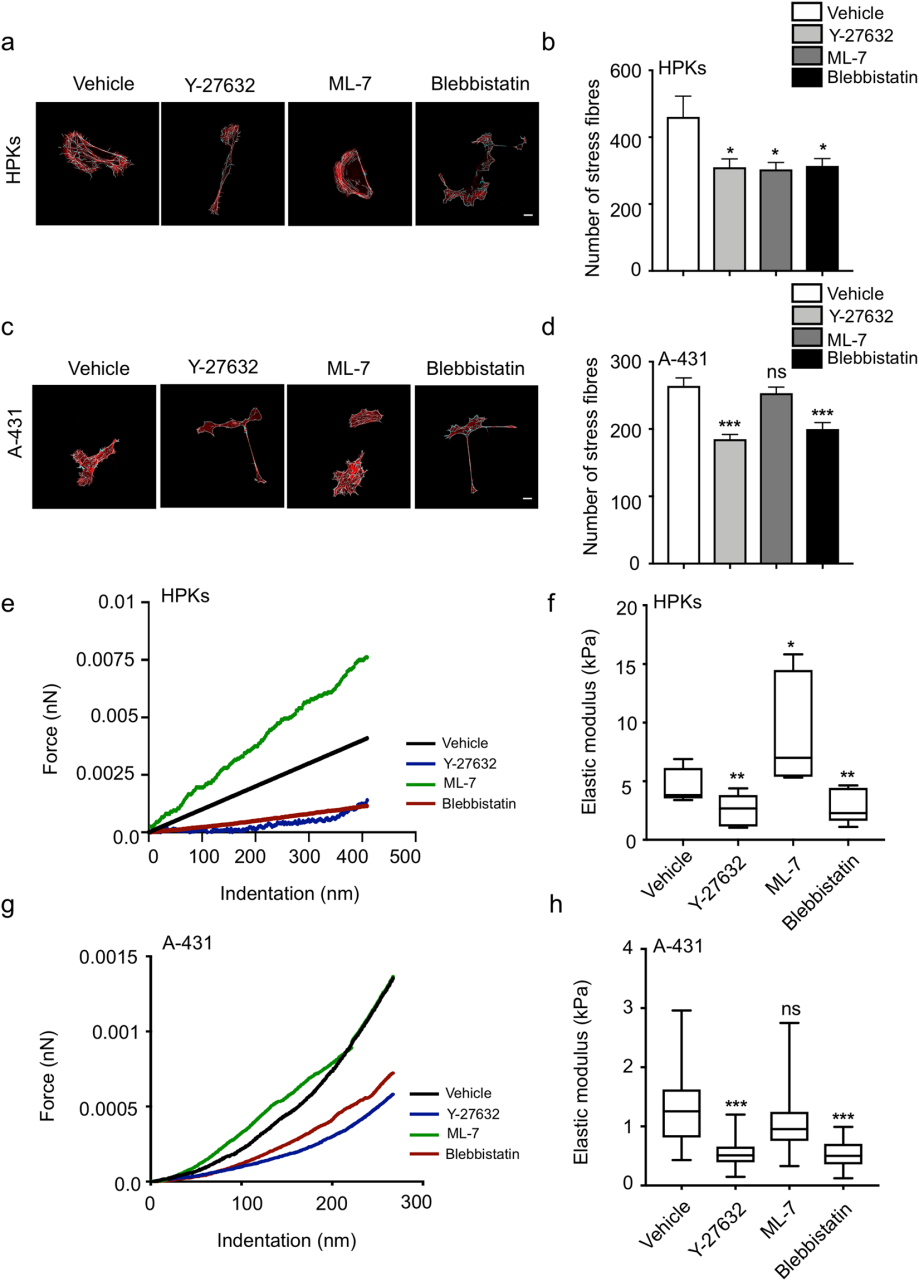
Role of ROCK and MLCK on actin stress fiber organization and cortical stiffness of HPKs and A-431: (**a**) Representative images of HPKs with the highlighted stress fibres. Magnification 63X. Scale bar 10 μm. (**b**) Quantification of the number of stress fibres (per cell) in HPKs upon treatment with inhibitors. n = 3. (**c**) Representative images of A-431 with the highlighted stress fibres. Magnification 63X. Scale bar 10 μm. (**d**) Quantification of the number of stress fibres (per cell) in A-431 upon treatment with inhibitors. n = 3. (**e,f**) Representative indentation curves obtained from the AFM experiment (**e**) and quantification (**f**) of the cortical stiffness of HPKs treated with ML-7, Y-27632 and Blebbistatin. n = 5. (**g,h**) Representative indentation curves (**g**) and quantification (**h**) of the cortical stiffness of A-431 treated with inhibitors. n = 3. Data is represented as Mean + SEM for bar graph. AFM data is represented as min to max box plot with median. Standard student’s t-test (two tailed) was performed *p < 0.05, **p < 0.01, ***p < 0.001, ns: not significant.

Actin stress fibres are important for the generation of contractile forces along with focal adhesions. We examined the roles of ROCK and MLCK on the organization of actin stress fibres and observed that inhibition of ROCK by Y-27632 treatment resulted in dissociation of the stress fibres and reduction in their numbers in both HPKs (Fig. 4a,b) and A-431 cells (Fig. 4c,d). ML-7 treatment resulted in reduction in number of stress fibres and peripheral actin localization similar to the focal adhesions in HPKs (Fig. 4a,b), with no effect on A-431 cells (Fig. 4c,d). Blebbistatin treatment led to the dissociation of actin stress fibres in both HPKs and A-431 (Fig. 4a–d). These data indicate that ROCK pathway majorly regulates the organization of actin stress fibres in HPKs and A-431 cells. However, MLCK regulates the actin stress fibre formation and organization in HPKs alone.

### Morphology of HPKs and A-431 is distinctly regulated by ROCK and MLCK

We observed that ROCK and MLCK differentially regulate the morphology of the cells as well. Inhibition of ROCK resulted in cells that were larger, more spread and more polar as compared to the vehicle treated cells with the formation of membrane protrusions in both HPKs and A-431. Inhibition of MLCK rendered the HPKs smaller, rounder and less polar but there was no significant change in morphology observed in A-431 cells. Blebbistatin treatment resulted in morphology similar to ROCK inhibition (Supplementary Fig. 1a–f). This suggests that ROCK plays a role in regulating cell morphology in HPKs and A-431. However, MLCK has a role in regulating cell morphology in HPKs only.

### ROCK and MLCK differentially influence the cortical stiffness of HPKs and A-431

Stiffness of cells is another biophysical characteristic which is highly correlated with the actin stress fibre organization and cellular morphology. Since ROCK and MLCK inhibition influenced the organization of stress fibres and morphology in HPKs and A-431, the roles of these kinases were examined on cell stiffness by atomic force microscopy (AFM). Cells were treated with the inhibitors and were indented using a pyramid-tipped probe. The elastic moduli of an individual cell were computed from the force curve obtained. It was observed that ROCK inhibition by Y-27632 significantly decreased the cortical stiffness of both HPKs (Fig. 4e,f) and A-431 (Fig. 4g,h), while ML-7 treatment increased the cortical stiffness HPKs alone (Fig. 4f). No effect was observed in A-431 cells upon MLCK inhibition (Fig. 4h). These data indicate that ROCK is crucial in maintaining the cortical stiffness of both HPKs and A-431.

### ROCK is the dominant kinase that phosphorylates MLC in A-431, whereas both ROCK and MLCK have distinct roles in regulating p-MLC in HPKs

**Figure 5:**
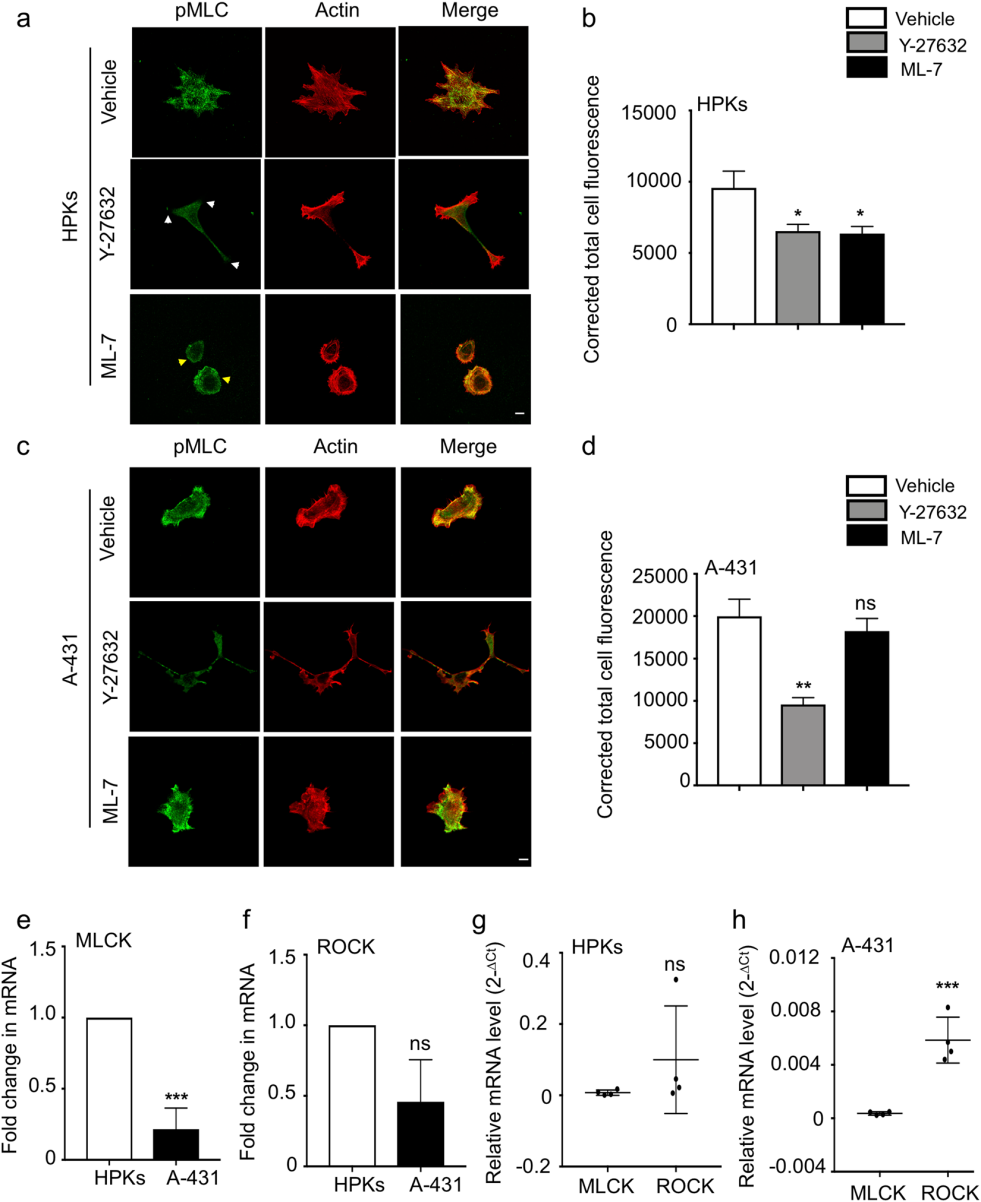
ROCK and MLCK expression regulates p-MLC in HPKs and A-431: (**a**) Representative images depict p-MLC (green) and actin (red) of HPKs. White arrows indicate loss of p-MLC at protrusion sites and yellow arrows indicate localization of p-MLC in the cell periphery. Magnification 63X. Scale bar 10 μm. (**b**) Quantification of the corrected total cell fluorescence of p-MLC in HPKs treated with inhibitors. n = 3. (**c**) Representative images depict p-MLC (green) and actin (red) of A-431. Magnification 63X. Scale bar 10 μm. (**d**) Quantification of the corrected total cell fluorescence of p-MLC in A-431 treated with inhibitors. n = 3. (**e**,**f**) Fold change in the mRNA levels of MLCK (**e**) and ROCK (**f**) compared between HPKs and A-431. (**g**,**h**) Comparison of the relative mRNA expression (represented as 2^−ΔCt^) of ROCK and MLCK in HPKs (**g**) and A-431 (**h**). n = 4. Data is represented Mean + SEM for bar graphs. Relative mRNA levels are represented as scatter plot with mean. Standard student’s t-test (two tailed) was performed *p < 0.05, **p < 0.01, ***p < 0.001, ns: not significant.

Since we observed that inhibition of ROCK and MLCK had distinct effects on the migration, contractility and focal adhesion organization of HPKs and A-431, we analysed the effect of ROCK and MLCK inhibition on the p-MLC levels in both cell types. p-MLC was examined by immunofluorescence using a primary antibody against the phosphorylated serine residue (S19) on MLC. We observed that blockade of ROCK in HPKs resulted in a notable absence of p-MLC in the actin rich protrusions sites (Fig. 5a, indicated by white arrows), though no effect was seen on the localization in A-431 (Fig. 5c). MLCK blockade resulted in localization of p-MLC at the periphery in HPKs (indicated by yellow arrows), while there was no difference in the localization of p-MLC in A-431 upon MLCK inhibition (Fig. 5a,c).

Quantification of the p-MLC level was performed by measuring the total corrected fluorescence from the immunofluorescence images. It was observed that ROCK inhibition resulted in significant loss of p-MLC in both HPKs (Fig. 5b) and A-431 (Fig. 5d). While MLCK inhibition also resulted in decreased p-MLC levels in HPKs (Fig. 5b), no significant difference was observable in A-431 upon MLCK inhibition (Fig. 5d). To ensure that the ML-7 used was active, different batches of the drug were used and were also tested on HeLa cells. ML-7 treatment resulted in a decrease in the p-MLC levels in HeLa cells (Supplementary Fig. 2a,b) in line with a previous observation^29^. These data suggest that ROCK, and not MLCK, is the dominant kinase responsible for the phosphorylation of MLC in A-431. Also, both ROCK and MLCK distinctly control the localization and levels of p-MLC in HPKs.

### MLCK levels are downregulated in A-431 as compared to HPKs

Since inhibition of MLCK did not have any effect on the p-MLC levels or any of the cellular characteristics tested in A-431 cells, we wished to examine if the lack of response to ML-7 (MLCK inhibitor) was due to alterations in the levels of MLCK in A-431. We compared the fold change in the mRNA levels of ROCK1 and overall MLCK between HPKs and A-431 by real time PCR. We observed that the mRNA levels of MLCK were downregulated in A-431 cells compared to the HPKs (Fig. 5e). There was an inconsistent difference in the mRNA levels of ROCK1 between HPKs and A-431 cells, which was not significant (Fig. 5f). We also compared the relative mRNA expression between ROCK1 and MLCK in HPKs and A-431.We observed that the relative mRNA expression of ROCK1 and MLCK was comparable in HPKs, whereas A-431 cells had a significantly higher level of ROCK as compared to MLCK (Fig. 5g,h). This indicates that the lack of cellular response to ML-7 treatment is due to the significant downregulation of MLCK in A-431 cells. The significantly higher levels of ROCK solely regulate contractile dynamics in A-431 cells.

### Phosphorylation of MLC is critical for cell migration and other associated cellular features

Our results with Y-27632 indicate that ROCK mediated p-MLC activity controls migration and other cellular features such as focal adhesions, stress fibre organization and morphology of A-431 cells. To strengthen our observations that phosphorylation of MLC is the key mediator of migration, we generated genetically modified A-431 cells with phosphomimetic MLC by replacing Ser19 and Thr18 with aspartic acid (DD-MLC-GFP). This mutant will therefore behave as constitutively phosphorylated p-MLC. Since Y-27632 inhibits the phosphorylation on Thr18 and Ser19 of MLC, addition of Y-27632 to cells transfected with DD-MLC-GFP should not have any effect on the migration or other cellular features, if they are regulated by p-MLC. As a control, we generated A-431 cells transfected with wild type MLC (WT-MLC-GFP), where Y-27632 treatment is expected to inhibit the p-MLC activity and migration of these cells, similar to non-transfected cells.

**Figure 6:**
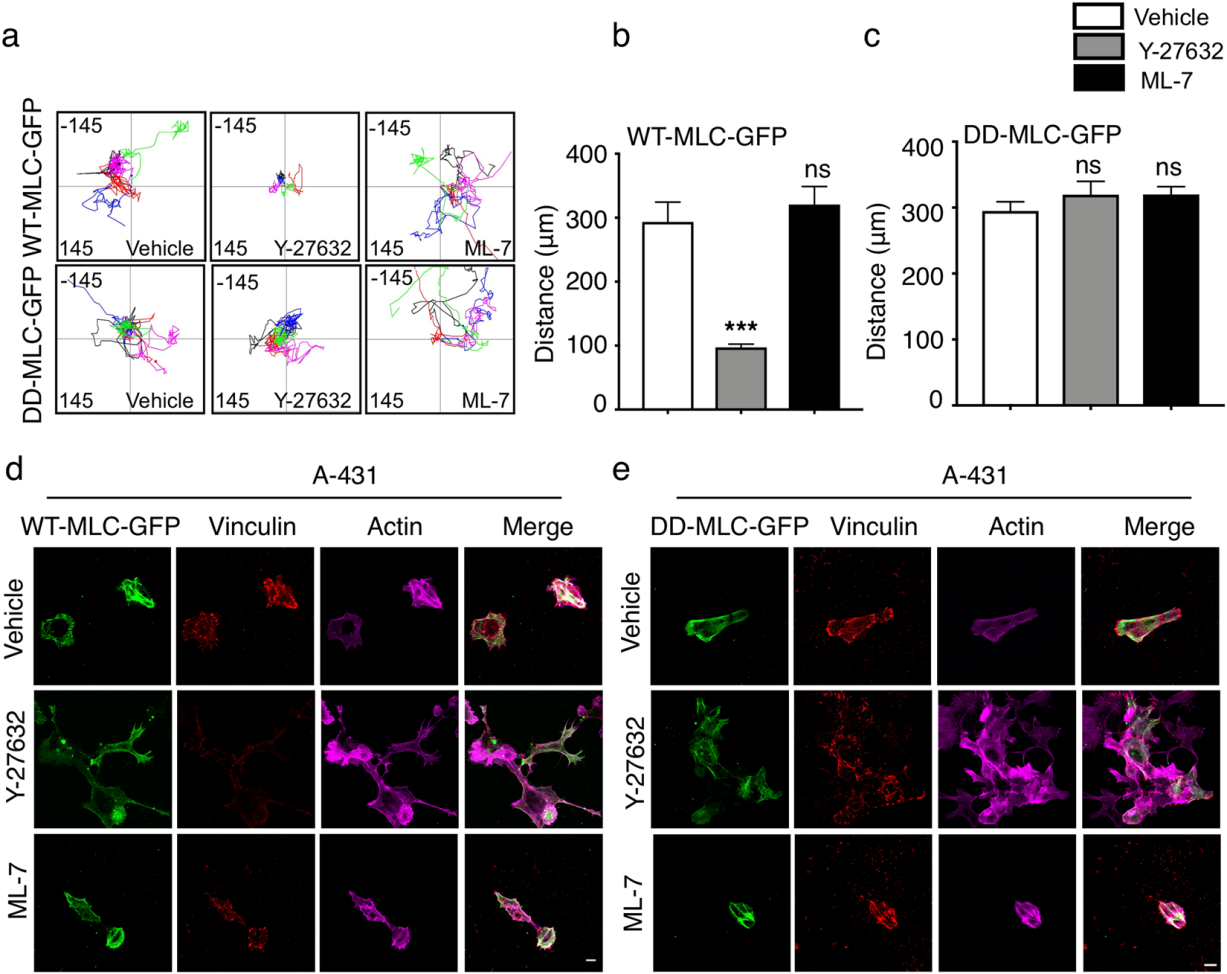
Phosphorylation of MLC by ROCK and MLCK is crucial for migration, focal adhesion, actin stress fibre organization. (**a**) Representative track plots of the migration of A-431 cells transfected with WT-MLC-GFP and DD-MLC-GFP. (**b**,**c**) Quantification of the total distance traversed and the velocity of A-431 cells transfected with WT-MLC-GFP (**b**) and DD-MLC-GFP (**c**). n = 3. (**d**,**e**) Representative images of MLC-GFP (green), vinculin (red) and actin (magenta) of A-431 cells transfected with WT-MLC-GFP (**d**) and DD-MLC-GFP (**e**). Magnification 63X. n = 2. Scale bar 10 μm. Data is represented Mean + SEM for bar graphs. Standard student’s t-test (two tailed) was performed ***p < 0.001, ns: not significant.

Similar to our observation with non-transfected cells, A-431 cells transfected with WT-MLC-GFP showed reduced migration upon Y-27632 treatment and no change by ML-7 (Fig. 6a,b). Y-27632 treatment also resulted in reduced focal adhesions, dissociation of stress fibres and more polar cells in A-431 cells transfected with WT-MLC-GFP (Fig. 6d) In contrast, ROCK inhibition by Y-27632 did not affect the migration (Fig. 6b) or the focal adhesion status, stress fibre formation and morphology of A-431 cells transfected with DD-MLC-GFP (Fig. 6d), indicating that ROCK mediated phosphorylation of MLC is critical for the migration, focal adhesion formation, stress fibre organization and morphology of A-431 cells.

## Discussion

Our study revealed few interesting observations regarding the role of ROCK and MLCK in regulating the actomyosin machinery, and ultimately the migration of human primary keratinocytes and epidermoid carcinoma cells: (1) ROCK is critical for controlling the biophysical properties (contractility, focal adhesions levels, stress fibre formation) of normal (HPKs) and malignant skin epithelial cells (A-431 cells) and impacts their migration by influencing the p-MLC levels. (2) In contrast, MLCK regulates the biophysical properties and migration of HPKs but does not have significant impact on A-431 cells due to negligible MLCK expression.

We observed that the migration of HPKs in a 3D environment was completely lost by the inhibition of ROCK as well as MLCK. Cell migration is a coordinated process involving the cytoskeleton and adhesion complexes^30^, which is regulated by MLC phosphorylation. Focal adhesions, in tandem with actin stress fibres generate the necessary forces required for the cell to migrate^31^. Focal adhesions are critical for the migration of cells in a dense matrix and reduction in focal adhesions is seen to adversely affect migration of cells in 3D^32^. In this study, Y-27632 treatment resulted in a complete loss of mature focal adhesions, as well as dissociation of actin stress fibres in both HPKs and A-431 cells. Such a cell will not be able to generate sufficient forces necessary for migration, as evident by the lack of retraction dynamics tested by the trypsin deadhesion assay (Fig. 2) and could be a contributing factor as to why Y-27632 treated cells show reduced migration in 3D as compared to the control cells. On the other hand, we observed that ML-7 treatment of HPKs, resulted in a decrease in both number and average area of the focal adhesions, along with a reduction in the stress fibres, which impacted contractility of the cells, resulting in a reduction in 3D migration as well. In contrast, ML-7 treated A-431 cells did not show any significant change in focal adhesions or stress fibres, which ultimately did not affect the 3D migration as well.

Studies have described that a balance of contractile force generation and cytoskeletal remodelling are essential for the efficient migration of cells in a 3D environment^33^. A loss in any of these attributes can impair 3D migration, as evident from the reduction in 3D migration due to the loss of contractile force generation by blockade of ROCK and MLCK in HPKs. Studies have reported that contractile force generation is of paramount importance for migration of cells in 3D^34,35^. We observed that p-MLC levels, which are crucial for contractile force generation, were also lowered in HPKs treated with both ROCK and MLCK inhibitors, resulting in abrogation in contractility and ultimately, decreased migration in both conditions. While inhibition of ROCK in A-431 cells led to a loss in 3D migration due to reduced p-MLC levels and contractility, MLCK inhibition did not affect these attributes of A-431 cells. This phenomenon seen in A-431 cells is also consistent with a previous study performed on melanoma cell line MDA-MB-453, where MLCK inhibition had no effect on the contractile dynamics of MDA-MB-453^13^. MLCK is known to be highly downregulated in cancer cells at both mRNA and protein levels^29^. We also observed that MLCK mRNA levels were highly downregulated in A-431 cells as compared to HPKs. The loss of MLCK in A-431 could be the major reason why treatment with ML-7 had minimal effects on the contractility and invasion of the malignant cells.

Phosphorylation of MLC by ROCK also regulated the focal adhesion organization and morphology of the cells as previously demonstrated^36^. Treatment of HPKs with ROCK and MLCK inhibitors resulted in the reduction of p-MLC levels, but the localization of p-MLC in the cells had varied effects on the focal adhesion organization as well as morphology of the cells. While ROCK inhibited HPKs showed an increase in polarity due to loss of p-MLC at the edges of the cell, MLCK inhibited cells showed localization of p-MLC in the cell periphery, resulting in rounder cells. The localization of focal adhesions and stress fibres in MLCK inhibited HPKs was also seen to be dependent on the p-MLC localization. Similarly, though there was no effect of MLCK inhibition in A-431, ROCK inhibition resulted in lack of mature focal adhesions, dissociation of stress fibres and highly polar cells due to loss of p-MLC. This was further confirmed when cells transfected with the phosphomimetic mutant of MLC did not show any alterations in focal adhesion organization or morphology as the inhibitors had no effect on the phosphorylation status of the constitutively phosphorylated (phosphomimetic) mutant, indicating that changes in p-MLC due to ROCK and/or MLCK inhibition were indeed responsible for the variations in focal adhesion organization, morphology and ultimately the migration of HPKs and A-431.

We also observed that MLCK and ROCK differentially regulate the stiffness of HPKs and A-431. Inhibition of MLCK significantly increased the cortical stiffness of HPKs, though there was a loss in the number of stress fibres. The increased stiffness could be due to the reduced cell spreading of the HPKs upon inhibition of MLCK, which increased the thickness of the cells. This phenomenon has been previously observed in hepatocytes as well^37^. Also, the presence of actin stress fibres at the cell periphery is known to reinforce cortical stiffness^38^. On the contrary, ROCK inhibition resulted in a significant decrease in the cortical stiffness of HPKs and A-431, due to depolymerisation of actin. The complete dissociation of stress fibres as well as increase in spread area due to the inhibition of ROCK resulted in decreased cortical stiffness, resulting in reduced 3D migration in both the cell types. Similar to the effect seen in this study, we have shown previously that abrogation of ROCK by Y-27632 treatment significantly reduced the stiffness in cancer cells^13^.

Our results from this study demonstrate important differences in the regulation of contractility and 3D migration by ROCK and MLCK between healthy and malignant skin cells. Our *in vitro* data about the expression levels is also supported by the fact that MLCK is seen to be down regulated in tumor samples of skin malignancies as well, analysed using TCGA database and Xena software (Supplementary Fig. 3a). In addition, analysis of the overall survival rate and MLCK expression revealed that low MLCK expression is associated with a slightly poorer prognosis as compared to high MLCK expression (Supplementary Fig. 3b). Developing biologics that target ROCK or upregulate MLCK may be of profound value in conditions where aberrant and increased invasion is seen in keratinocytes, such as in pathological conditions like keratinocyte cancers and inflammation. It is also of importance to understand the influence of various upstream and downstream signalling molecules of ROCK in important cellular functions of keratinocytes, including normal and pathogenic conditions such as wound healing, tissue repair, inflammation and cancer. Apart from MLC and MLC phosphatase, ROCK also phosphorylates LIMK, which in turn phosphorylates Cofilin and regulates the actin de-polymerization. Studies have associated LIMK and Cofilin with higher invasion potential of several malignant tumours^39,40^. Although ROCK and p-MLC are the terminal regulators of this pathway along with intermediary effectors (LIMK/Cofilin), NMMIIA has been shown to be predominant in generating cellular contractility, regulating actin dynamics and cell adhesion^6,41^. A comprehensive study demonstrating the role of the individual components of the ROCK and MLCK pathway can provide a better insight on how these act in tandem to generate contractile forces in various cell types and physiological conditions.

## Materials and Methods

### Keratinocyte isolation and cell culture

All experimental protocols were approved by the IIT Bombay Institute Ethics committee and Ethics committee for academic research projects, T.N. Medical College and BYL Nair Ch. Hospital, and were carried out in accordance with the relevant guidelines and regulations. HPKs were isolated from leftover samples of cosmetic surgery as described previously^42^, with the informed consent of the participants. The skin was incubated overnight in 2.4 U dispase II (Roche, Mannheim, Germany) at 4 °C. The epidermis was separated and trypsinised (0.25% Trypsin-EDTA, Himedia, India) for 20 minutes at 37 °C. The cell suspension was filtered through a cell strainer (40 μm) and washed twice in neutralizing medium (10%FBS). The single-cell suspension obtained was maintained in serum-free Epilife Keratinocyte Growth Medium (Gibco, USA) and supplemented with Epilife defined growth supplements (HKGS, Gibco, USA) at 37 °C in a humidified incubator with 5% CO_2_. HPKs in passages 2 to 5 were used for all experiments.

A-431 (Human epidermoid carcinoma) and HeLa (cervical carcinoma) cell lines were obtained from National Centre for Cell Sciences (NCCS) cell repository, Pune, India. Cells were cultured in Dulbecco’s Modified Eagle’s Medium with glucose (Sigma, Germany) containing 10% FBS, 1% penicillin-streptomycin antibiotic solution and 1 mM sodium pyruvate (Gibco, USA) at 37 °C in a humidified incubator with 5% CO_2_.

### Preparation of collagen coated coverslips

Circular glass coverslips were sterilized with 70% ethanol and UV treatment and incubated with Rat tail collagen type I (5 μg/cm^2^) (Gibco, USA) at 4 °C overnight and washed thrice with 1X DPBS. Cells were seeded on the collagen coated coverslips for all experiments.

### Treatment with inhibitors

ML-7 (MLCK inhibitor), Y-27632 (ROCK inhibitor) and Blebbistatin (global myosin inhibitor) (Calbiochem, USA) were used at a final concentration of 10 μM and treated for 2 hours for experiments, unless specified otherwise. DMSO was used as the vehicle control.

### 3D migration assay

24 well plates were treated with 2% glutaraldehyde for 20 minutes and washed thrice with sterile distilled water. Collagen gels (final concentration 1 mg/ml) were prepared using rat tail collagen type I. 1 N NaOH was used to adjust the pH to 7.4. Cell suspensions with appropriate cell density were mixed with the collagen gel and immediately seeded on glutaraldehyde coated wells. The gels were allowed to solidify for 1 hour at 37 °C and were treated by layering media with the treatments (20 μM) on the gels. The cells were imaged using a spinning disc confocal microscope (Zeiss, Germany) under 10X magnification for 24 hours. The migration of the cells was tracked using ImageJ software.

### Trypsin deadhesion assay

The treated cells were washed with 1X DPBS and pre-warmed trypsin (0.25%, 500 μl) was added to the well. Images were captured every 3 seconds till the cells rounded up. The normalized change in area ($\bar{A}$) was calculated using the formula:

$$\bar{A}=\frac{{A}_{i}-{A}_{t}}{{A}_{i}-{A}_{f}}$$

where *A*_*i*_ is cell area at time t = 0, *A*_*t*_ is area at time = t, and *A*_*f*_ is area at the final time point. The de-adhesion curves were fitted with the Boltzmann equation to obtain the time constants **τ**_1_ and **τ**_2_:

$$(\bar{A}=\frac{1}{1+{e}^{(t-{\rm{\tau }}1)/{\rm{\tau }}2}})$$

**τ**_total_ was calculated as the sum of **τ**_1_ and **τ**_2_. The analysis was done using ImageJ software and the deadhesion curves were fit using Graphpad Prism 7 software.

### Immunofluorescence

For the p-MLC staining, treated cells were fixed with 4% paraformaldehyde for 20 minutes, permeabilized using 0.1% Triton X-100 for 4 minutes, blocked with 2% BSA for 30 minutes and incubated with anti p-MLC antibody (Invitrogen, USA) (1:100) overnight at 4 °C. The cells were incubated with Alexa Fluor 488 goat anti-rabbit secondary antibody (Invitrogen, USA) and Alexa Fluor 647 Phalloidin for 2 hours. Images were acquired using LSM confocal microscope (Zeiss, Germany) under 63X magnification. The corrected total cell fluorescence (CTCF) obtained after background subtraction from the formula: Integrated cell density – (Mean fluorescence of the background*Area of the cell).

For focal adhesion staining, the treated cells were permeabilized using a 1:1 solution of cold 4% paraformaldehyde and 0.1% Triton X-100 solution for 5 minutes on ice. The cells were incubated in 4% paraformaldehyde for 1 minute on ice, washed thrice and blocked with blocking solution (1.5% BSA and 0.1% Triton X-100 in DPBS) for 30 minutes on ice and incubated with anti-vinculin antibody (Invitrogen, USA) overnight. The cells were incubated with Alexa Fluor 488 goat anti-mouse secondary antibody and Alexa Fluor 647 Phalloidin for 2 hours. Images were acquired using LSM confocal microscope (Zeiss, Germany) under 63X magnification. The quantification of the number and size of the focal adhesions was performed using the image processing tools and threshold feature of ImageJ software as previously described^43^.

For actin stress fibre imaging and quantification, Alexa Fluor 647 Phalloidin stained cells were imaged at 63X magnification using a Laser scanning confocal microscope (Zeiss). The quantification was done using the FilamentSensor software as previously described^44^.

### Analysis of cell morphology

Treated cells were fixed and stained with Alexa Fluor 647 Phalloidin (Invitrogen, USA) as mentioned above. Images were acquired using a spinning disc confocal microscope (Zeiss, Germany) under 10X magnification. The area and circularity of the cells was calculated using ImageJ software.

### Atomic force microscopy (AFM)

AFM measurements were performed with an Asylum MFP3DAFM (Asylum Research, CA) coupled to an epifluorescence microscope (Nikon, Japan). Individual treated cells were indented using a pyramid-tipped probe with a nominal spring constant of 25 pN/nm. The first 1 μm of force-indentation curves was fitted with the Hertzian model for a pyramidal tip to obtain the elastic modulus. The analysis of the indentation curves was performed using MATLAB software as described previously^45^.

### RNA isolation and real time PCR

RNA was extracted from A-431 cells and HPKs using the RNeasy Plus Mini Kit (Qiagen, Germany). Reverse transcription was performed using the Quantitect Reverse Transcription kit (Qiagen, Germany) with 1 μg of RNA. cDNA levels were quantified by SYBR Premix Ex2 kit (Tli Plus) (Takara, Japan) by StepOnePlusTM Real- time PCR System (Applied Biosystems, USA). Pre-designed primers (Kiqstart primers) for ROCK1 (Forward primer: 5′ CAAGGTGGTGATGGTTATTATG 3′, Reverse primer: 5′ TATCACCTACAAGCATTTCG 3′ and MLCK (total MLCK; (Forward primer: 5′ AGAATCTGAAGATGTGTCCC 3′, Reverse primer: 5′ ATCTTGCAGTCAAATCTAGC 3′) were obtained from Sigma, Germany. Quantification of the relative gene expression was performed using StepOneTM Software v2.3 (Applied Biosystems, USA). GAPDH was used as the endogenous control.

### Transfection of cells with plasmids

pEGFP-MRLC1-WT (Addgene plasmid #35680; http://n2t.net/addgene:35680; RRID: Addgene_35680) and pEGFP-MRLC1T18D, S19D (Addgene plasmid #35682; http://n2t.net/addgene:35682; RRID:Addgene_35682) were gifts from Tom Egelhoff^46^. A-431 cells were transfected with the plasmids using Lipofectamine 3000 (Thermo Fisher Scientific, USA) according to the manufacturer’s protocol.

### Statistical analysis

Statistical analysis was performed using the standard student’s t-test (two tailed). p value less than 0.05 was considered to be statistically significant. The extent of significance was designated as *p < 0.05, **p < 0.01, ***p < 0.001.

## Supplementary information

Supplementary information 

## Data Availability

The authors confirm that the data supporting the findings of this study are available within the article [and/or] its supplementary materials.
