# Supplementary material for: Blockade of ROCK inhibits migration of human primary keratinocytes and malignant epithelial skin cells by regulating actomyosin contractility
Source: Sci Rep. 2019 Dec 27;9:19930. doi: 10.1038/s41598-019-56447-2 (PMC6934852; doi:10.1038/s41598-019-56447-2)
Supplement: Supplementary file 1 — Supplementary information [file 41598_2019_56447_MOESM1_ESM.pdf]

Blockade of ROCK inhibits migration of human primary keratinocytes and malignant epithelial skin cells by regulating actomyosin contractility

Srisathya Srinivasan[1], Sreya Das[1], Vishakha Surve[1], Ankita Srivastava[1], Sushant Kumar[1], Nikita Jain[1], Abhijeet Sawant[2], Chitra Nayak[3] and Rahul Purwar[1]*

[1] Department of Biosciences & Bioengineering, IIT Bombay, Mumbai, Maharashtra, INDIA

[2] Department of Plastic Surgery, Topiwala National Medical College & BYL Nair Charitable Hospital, Mumbai, Maharashtra, INDIA

[3] Department of Skin and Venereal Diseases, Topiwala National Medical College & BYL Nair Charitable Hospital, Mumbai, Maharashtra, INDIA

*Corresponding author

Dr. Rahul Purwar

Associate Professor

Department of Biosciences & Bioengineering,

IIT Bombay,

Mumbai, Maharashtra, INDIA

Phone: +912225767737

Email: purwarrahul@iitb.ac.in

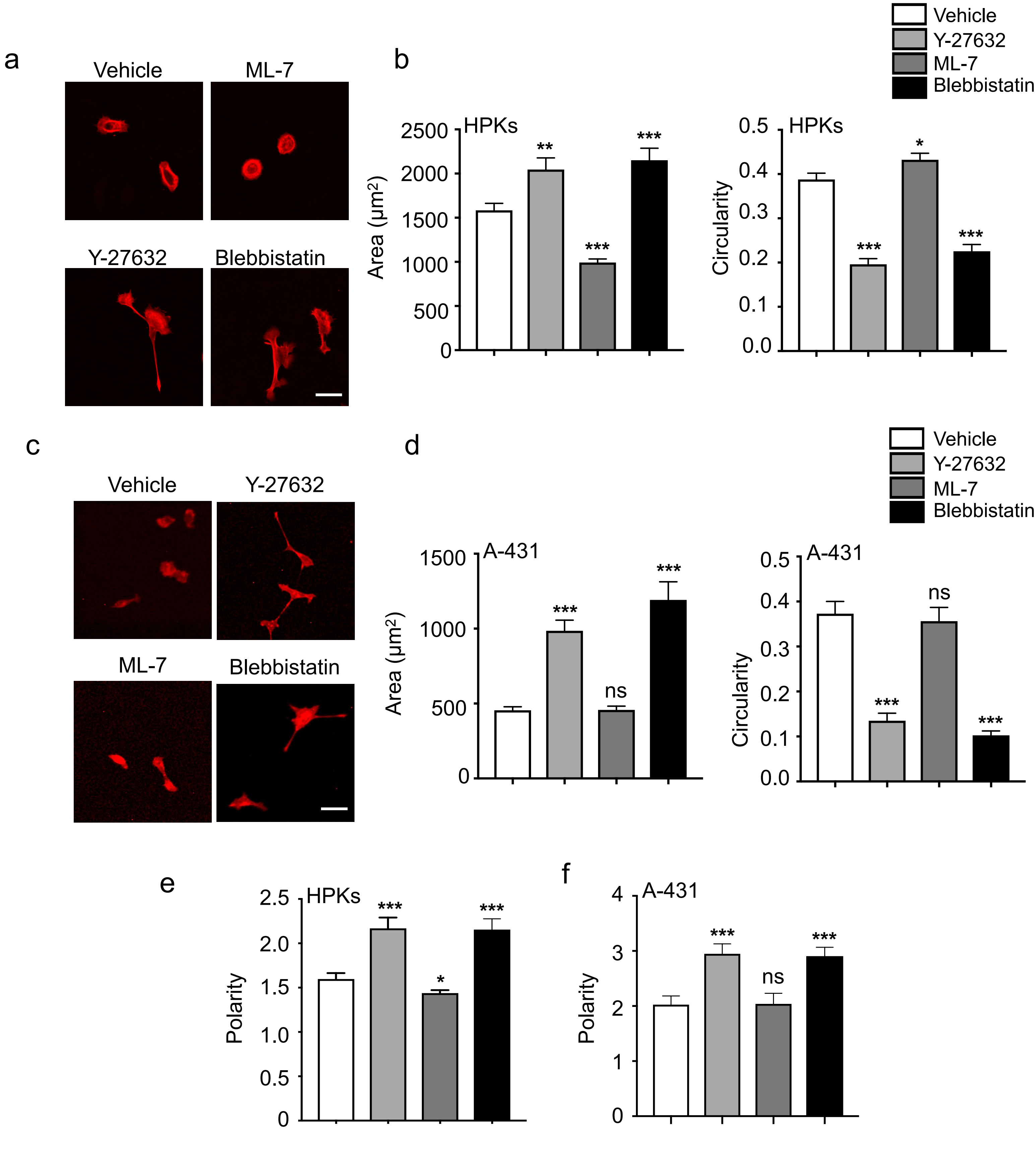

Supplementary Figure 1: ROCK and MLCK distinctly regulate morphology of HPKs and A-431: (a) Representative images of fluorescently tagged phalloidin stained HPKs treated with inhibitors. Magnification 10X. Scale bar 50µm. **(b)** Quantification of the area and circularity of HPKs treated with inhibitors. n=3. **(c)** Representative images of A-431 stained with fluorescently tagged phalloidin treated with inhibitors. Magnification 10X. Scale bar 50µm. **(d)** Quantification of the area and circularity of A-431 treated with inhibitors. n=3. **(e)** Quantification of the polarity of HPKs treated with inhibitors. **(f)** Quantification of the polarity of A-431 treated with inhibitors**.** n=3. Data is represented as mean + SEM for the bar graph.* $p<0.05$, ** $p<0.01$, ***$p<0.001$, ns: not significant.

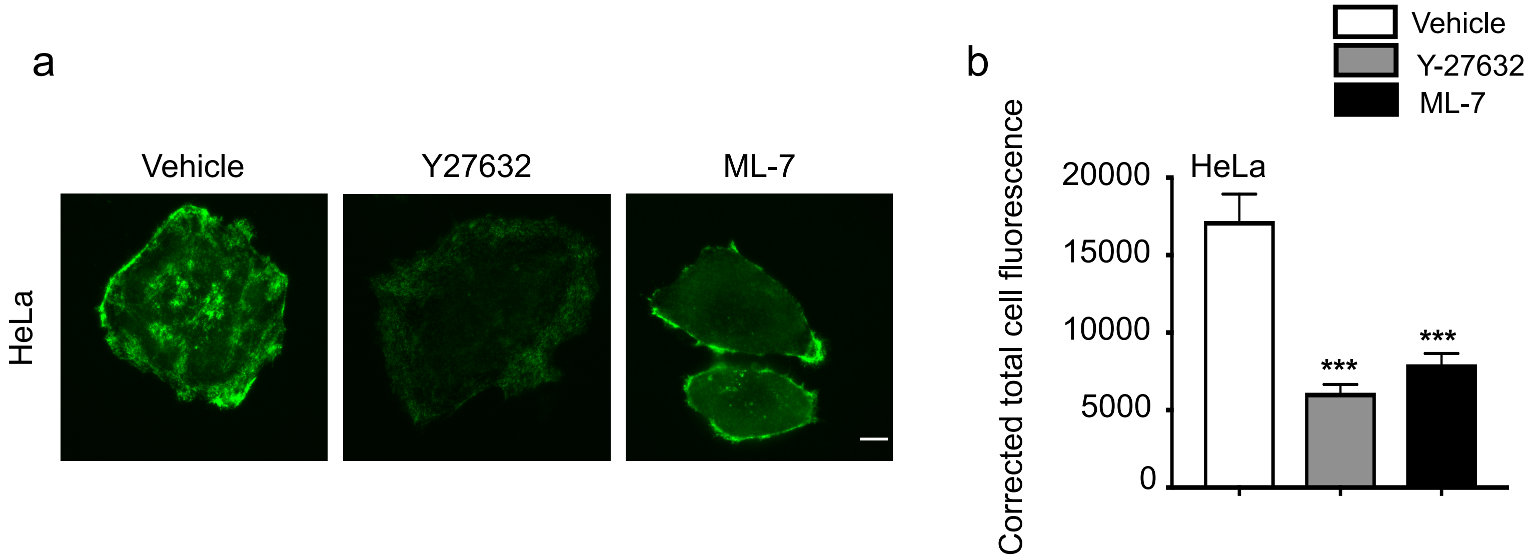

Supplementary Figure 2: ROCK and MLCK regulate p-MLC levels in HeLa cells: (a) Representative images of p-MLC of HeLa cells treated with inhibitors is shown. Magnification 100X. Scale bar 10µm. **(b)** Quantification of the corrected total fluorescence indicative of p-MLC levels in HeLa cells treated with inhibitors. Bar graphs are represented as mean+ SEM. *** $p < 0.001$.

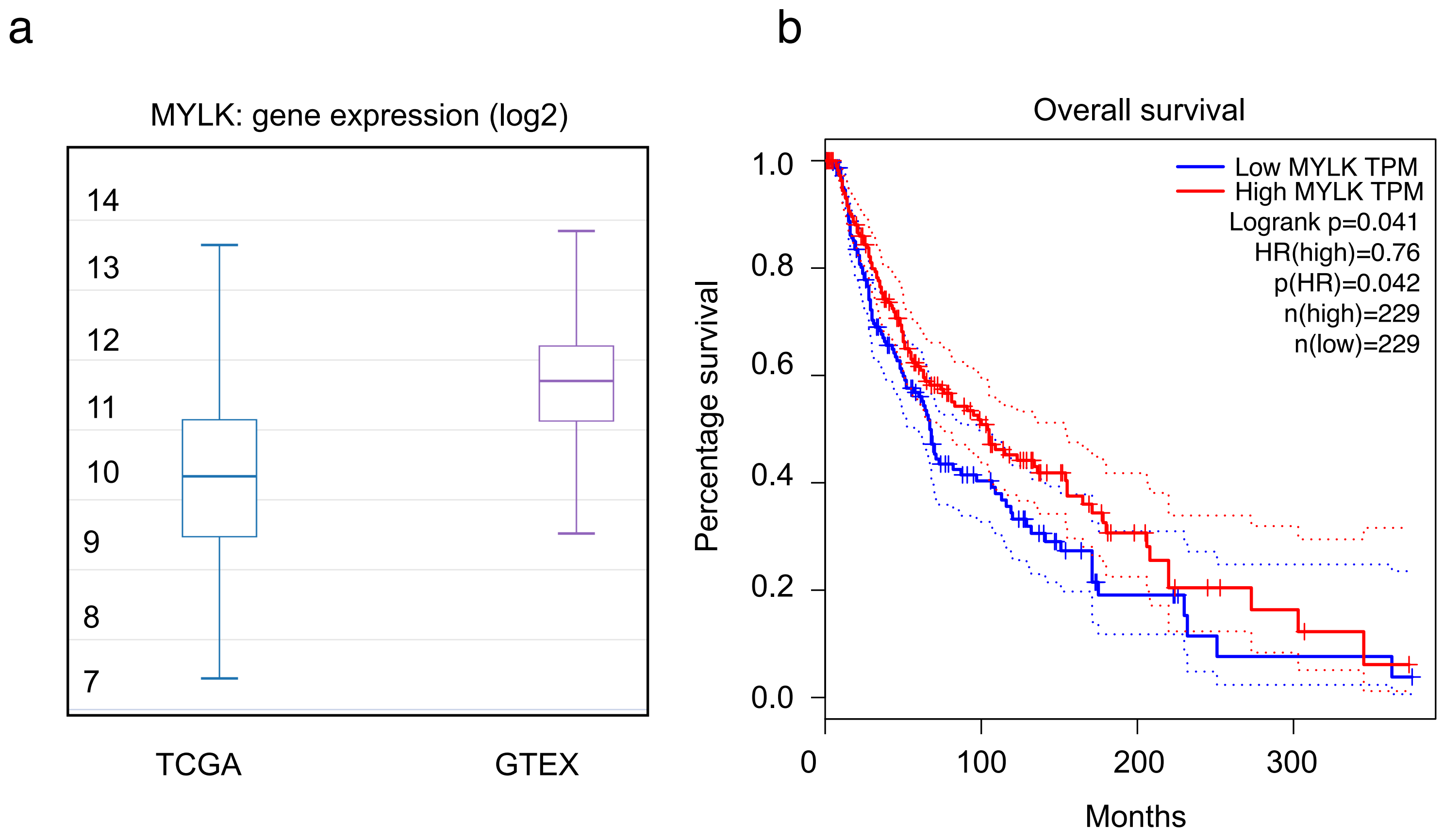

Supplementary Figure 3: Lower MLCK levels are associated with decrease in overall survival: (a) Levels of gene expression of MLCK (MYLK gene) as compared between tumor samples from TCGA database and normal samples from GTEX database of 1250 samples. **(b)** Comparison of the overall survival rate in patients with skin cancers with high and low levels of MLCK expression (MYLK gene)
